# Commercially Available Blue Diode Laser Engraver Operating at 455 nm as an Affordable LD-REIMS Ionization Source

**DOI:** 10.1021/acs.analchem.5c00724

**Published:** 2025-04-30

**Authors:** Prisca Weider, Daniel Heffernan, Min Qiu, Marco Klein, Carl Witthöft, Wei Chen, Nicole Strittmatter

**Affiliations:** Department of Biosciences, School of Natural Sciences, Technical University of Munich, Garching b. München 85748, Germany

## Abstract

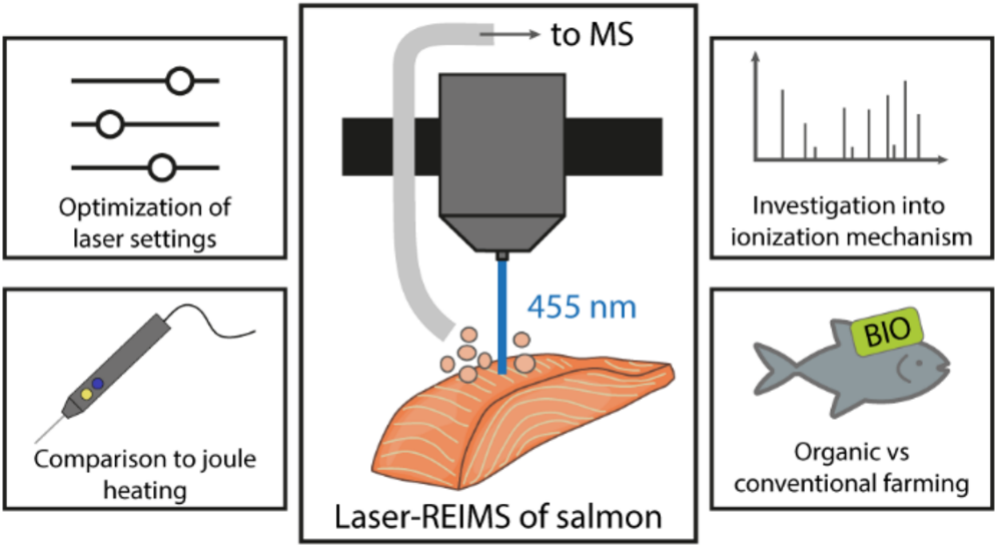

Lasers are commonly used for mass spectrometric applications to perform laser ablation–desorption and ionization; however, the use of visible light is not very common. Here, we report a commercially available visible light laser engraver operating at 455 nm as an ionization source, generating rich spectral profiles featuring predominantly lipid species, such as fatty acids and glycerophospholipids. Laser settings such as the speed of movement over the sample and laser power were tested, resulting in an optimum laser speed of 300 mm/min and a laser power of 30–50% for the analysis of fresh salmon tissue samples. Spectra generated were found to be similar to those produced by a conventional REIMS mechanism using Joule heating of the tissues, which was consolidated by comparative studies of the ion formation mechanism. The generated spectra show a slightly higher signal in the lower mass range, suggesting a higher degree of in-source fragmentation; however, no spectral feature was unique to either method. To test the suitability of the visible laser system to act as an REIMS-like profiling technique for food authenticity testing, we assessed the discrimination of Norwegian farmed salmon samples (*n* = 26) produced using conventional and organic farming methods.

## Introduction

The application of visible lasers for ion generation in mass spectrometry (MS) is not widespread as visible light is not strongly absorbed by most biomolecules.^1^ This suggests the use of a matrix to absorb the photons, and indeed most work in this field has been performed for applications using matrix-assisted laser desorption ionization (MALDI), which if performed with visible lasers is termed visible MALDI or vis-MALDI. The majority of MALDI applications are performed with lasers in the ultraviolet (UV) or mid- to far-infrared (IR)-range, with the most frequently used wavelengths being 337 nm (N_2_ laser) and 355 nm (frequency tripled Nd/YAG laser) in the UV range and 2.94 μm (Er/YAG laser) and 10.6 μm (TEA-CO_2_ laser) in the IR range. This is partially due to the large variety of suitable matrix compounds that can fulfill the requirements to absorb laser energy as well as facilitate ionization when desorbed with the analyte. However, not many compounds are known that can perform both roles in the visible light range.^2^ Binary matrix mixtures have been used to overcome this problem, in which one compound absorbs the energy and the second compound promotes ion formation. The absorption of visible laser light can also be promoted through small particles or surface structures, as is deployed in surface-assisted laser desorption/ionization (SALDI). Early studies included the use of red dyes such as Rhodamine and Neutral Red as possible matrices for visible MALDI, either pure or in binary liquid mixtures (for use with 532 nm lasers).^3−5^ Similarly, SALDI was used with a 532 nm laser to analyze biomolecules codeposited onto a graphite surface with glycerol.^6^ Hu et al. performed visible MALDI using coumarin dyes at a wavelength of 480 nm to detect peptides, noting softer ionization patterns than UV-MALDI, but also voicing a preference for use of green 532 nm lasers.^7^ Sun et al. showed atmospheric pressure visible MALDI, while Chen et al. used a gold nanorod substrate for LDI analysis, again both studies deploying 532 nm lasers.^4,8^ Niehaus et al. found visible MALDI feasible only with 2-(4-hydroxyphenylazo)benzoic acid (HABA) and a tunable laser operating in the violet range between 380 and 420 nm.^9^

The application of a matrix, however, complicates the analysis of low molecular weight molecules due to matrix interferences (clusters and matrix fragments), which continue to drive interest in matrix-free laser desorption ionization (LDI). This is, however, generally associated with a harder ionization mechanism, leading to most LDI studies opting for lasers in the IR range that transfer less energy to the analytes than UV lasers. Widespread laser ablation inductively coupled plasma (LA-ICP-MS) works with lasers in the deep UV range (such as 193 and 213 nm); however, fragmentation is not a concern due to the following atomization in the ICP. Other matrix-free techniques include ELDI (mid-IR lasers and UV lasers at 337 and 266 nm),^10,11^ LAESI (2.94, 10.6 μm),^12−14^ LD-REIMS^15,16^ (3, 10.6 μm), and the SpiderMass technology (Er/YAG at 2.94 μm).^17^

Rapid evaporative ionization mass spectrometry (REIMS) is an ambient mass spectrometry technique that was initially developed for the in situ, in vivo tissue identification in intrasurgical settings.^18−20^ While initially primarily used with electrothermal energy devices deploying Joule heating mechanisms, a range of other energy tools such as Cavitron ultrasound aspiration (CUSA) and laser-based tools have also been shown to give comparable spectra.^18,21^ Schäfer et al. have tested a range of different lasers for their suitability in REIMS, ranging from ultraviolet (UV) to the infrared (IR) wavelength range (266, 337, 355, 532, 785, 1064 nm, and 2.94 and 10.6 μm including surgical CO_2_ laser, Nd/YAG and N_2_ lasers, diode and OPO lasers). They observed that practically uniform spectra were obtained with lasers operating at 266, 337, and 355 nm and 2.94 and 10.6 μm. However, no spectra were detected at 532, 785, or 1064 nm, which was associated with biological tissues exhibiting the lowest absorbance and instead optical scattering as primary interaction in the 400–1200 nm range.^22^

With this study, we tested a commercially available, cheap laser engraver with a blue diode laser (455 nm, 20 W maximum laser power, costs approximately 700 €) for its applicability to serve as a laser ablation and ionization source for mass spectrometry applications in the food authenticity field. Especially, the IR-based lasers rely on the water O–H vibrational bands for ionization; water, however, has its near minimum absorbance in the blue range. Yet other tissue constituents still feature absorption in the blue range, such as bilirubin, oxy- and deoxyhemoglobin, and lipids.^23^ The advantages of the tested setup include low purchasing costs, making it widely affordable. Furthermore, commercial laser engravers generally comprise a programmable two-dimensional (2D) automated moving stage with unmatched range of movement (40 × 40 cm^2^ in our case) with intuitive, freely available software solutions, very fast scan speeds (up to 200 mm/s), and feature size as low as 100 μm (with engraving accuracy in the range of 0.01 mm). This makes these setups attractive for high-throughput yet budget-friendly profiling applications. The engraver model we acquired already has an integrated aerosol transfer system, which we modified to allow connection to the mass spectrometer. We used salmon as a sample system as it is a well-studied and widely consumed commodity, which, however, comes with concerns regarding food authenticity and mislabeling. Thus, fast and cost-efficient, automatable authentication tools to detect food fraud, including mislabeling (of species, country of origin, or organic growth conditions) and inferior quality products, are needed.

## Experimental Section

### Samples and Chemicals

Fresh and frozen farmed salmon samples were acquired in local supermarkets in the greater Munich metropolitan region in the spring of 2024. Only samples from European aquaculture (a total of *n* = 24) were included for the experiment comparing the laser and the Joule heating REIMS mechanism. A second sample set from Norwegian aquaculture only was used for the analysis of the rearing conditions (*n* = 13 conventional and *n* = 13 organic specimen). A full list can be found in Supporting Tables 1 and 2.

### Description of Laser Engraver and Software

Analysis was performed using an Atomstack A20 laser engraver and cutter (purchasing costs varying with supplier and between 650 and 800 EUR) equipped with a diode laser operating at a wavelength of 455 ± 5 nm and 20 W output power, achieved by coupling four individual 5 W diode lasers. The maximum speed is 12,000 mm/min with a working range of up to 40 × 40 cm^2^. The laser head is equipped with an integrated aerosol transfer line that is connected to an air assist module to avoid excessive smoke accumulation. We have removed the air assist module and used the aerosol line to contain a 1/8 in o.d. PTFE tubing, guiding it close to the ablation area. An SMC (Tokyo, Japan) ZH series body ported-type vacuum ejector with 0.7 mm nozzle nominal size (minimum pressure reached −90 kPa, maximum suction flow rate 12 L/min) has been installed 80 cm from the laser and 125 cm from the MS inlet to transport the aerosol from the mass spectrometer for analysis. A 1/16 in o.d. metal rod was pierced through the end of the 1/4 in polymer transfer tubing approximately 2 mm before the end and placed in front of the opening of the inlet capillary of the MS (approximately 2 mm distance) to avoid excessive contamination of the MS instrument through particulate matter. The distance between the sample surface and the laser exit was 4.5 mm, in line with manufacturers’ recommendations and controlled through a manufacturer-provided guide tool. Unless stated otherwise during optimization experiments, the laser was operated at a power setting of 30% and a speed of 500 mm/min. A scan pattern of 1 line/mm was used to generate line scans on the sample.

### REIMS Analysis

A monopolar electrosurgical handpiece with a needle electrode connected to an Erbe Elektromedizin (Tübingen, Germany) ICC350 electrosurgical generator was used to perform REIMS analysis relying on a Joule heating mechanism. A PTFE aspiration and aerosol transfer line was used to transport the generated aerosol to the MS inlet. Unless stated otherwise, aerosol transfer was aided by a Venturi pump analogous to the laser setup. REIMS analysis was performed using auto cut mode, program 0, effect 2, and a max power output of 50 W.

### Exactive MS Instrument Parameters

Mass spectrometric analysis was performed in negative ion mode using a Thermo Scientific Exactive classic instrument. Capillary temperature was 250 °C, capillary voltage was set to −45 V, skimmer voltage to −30 V, and tube lens voltage to −140 V. An injection time of 500 ms, ultrahigh resolution (*R* = 100,000 at 200 *m*/*z*), and an AGC target: 3 × 10^6^ (high dynamic range) were used.

### Data Analysis

Principal component analysis was performed in Matlab R2022b following data preprocessing with the maize script (James McKenzie, Imperial College London) using a data bin size of 0.001 Da, total ion count (TIC) normalization and log transformation, and alignment following two-point data recalibration (to *m*/*z* 699.4606 and 885.5499). For supervised analysis, partial least-squares discriminant analysis (PLS-DA) was performed on the extracted ion intensities through the MATLAB *plsregress* function. Data standardization was conducted using the *zscore* function. The classification model was built and evaluated by leave-sample-out cross-validation with a maximum component number of 10. If cross-validation determines the optimal number of components to be 1, then 2 components will be used to help understand the data structure for interpretation and visualization purposes. Model performance was assessed quantitatively by constructing confusion matrices for selected component setups. Least absolute shrinkage and selection operator (LASSO) regression was also performed in MATLAB, using the *lasso* function with 1SE lambda selection criterion (cross-validation specification = 10). Lists of specific features (*m*/*z* values) and receiver operating characteristic (ROC) curves were generated for each class. The remaining data analysis was performed by using QualBrowser and Excel.

### Fragmentation Analysis

The data-dependent fragmentation was performed on a Q-Exactive Plus instrument using the laser setup as described earlier and comparable instrument parameters (S-Lens setting was 100, resolution in full scan mode 70,000, resolution in MS/MS mode 17,500, Injection time was 150 ms). Data annotation was performed manually using QualBrowser.

### Studies into Ionization Mechanism

Staedtler Lumocolor blue, red, yellow, and purple, as well as Edding 400 red, were deposited onto the API building blocks of a Thermo LTQ XL instrument in positive ion mode. After re-evacuation, salmon samples were analyzed using monopolar REIMS and the blue laser as described above.

### Safety Considerations

The emission of visible light with a beam power of 0.5 W or more classifies the laser used as class 4, meaning that it poses a severe hazard to both the eyes and skin. Appropriate personal protective equipment (PPE), in particular, laser safety goggles to filter the specific wavelength, is necessary. The laser should be housed within a controlled environment to prevent accidental exposure by bystanders entering the area. Proper ventilation to disperse fumes and particulates generated during the operation is recommended.

## Results and Discussion

### Suitability of a Blue Laser as REIMS Source

**Figure 1:**
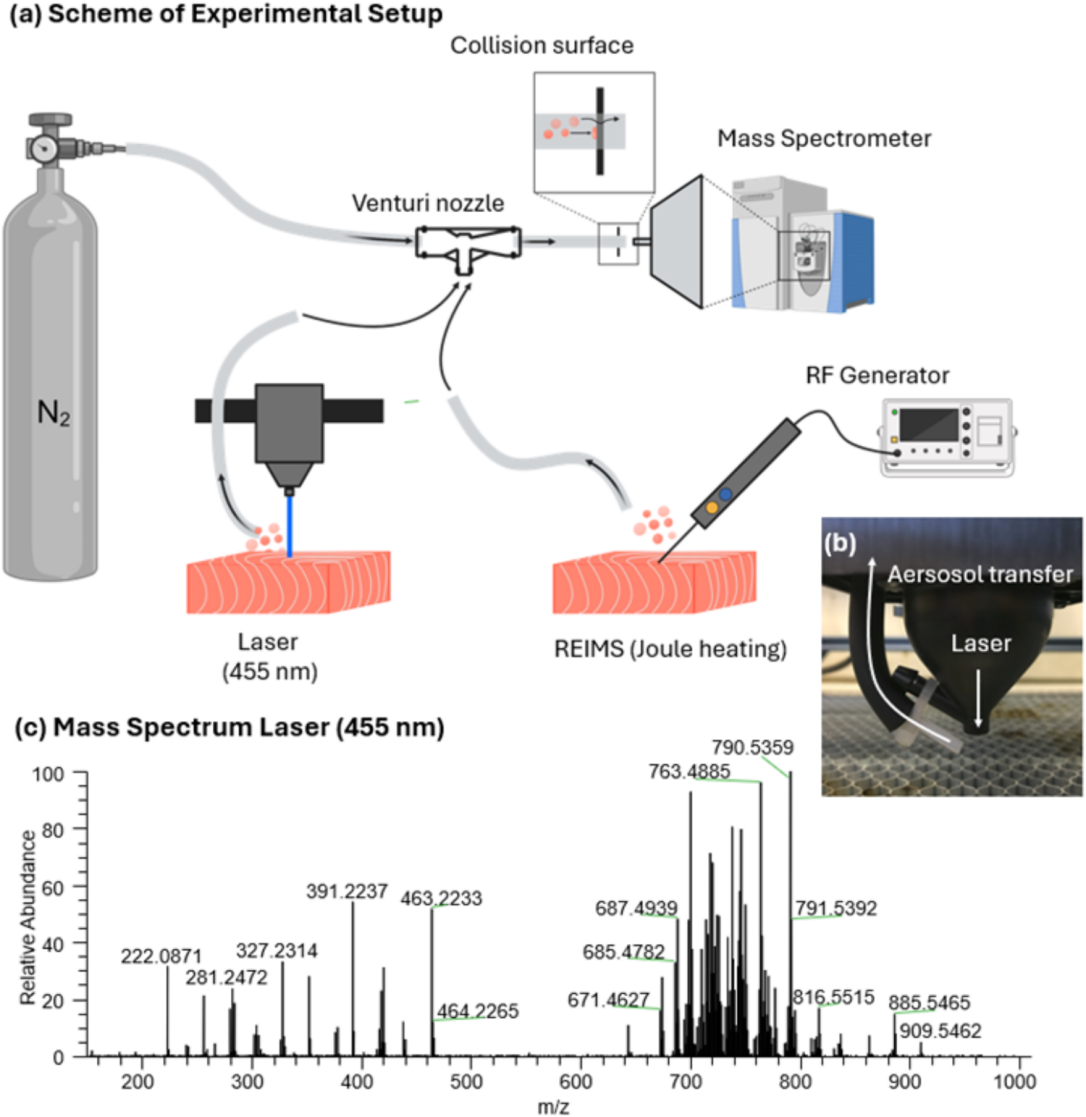
(a) Experimental setup for REIMS and laser engraver deploying the Venturi effect for aerosol transport and introduction into MS. (b) Shows a photograph of the sampling head. (c) Resulting spectra from laser irradiation at 455 nm (see Figure S1 for comparison to REIMS spectra). Created partially in BioRender. Heffernan, D. (2025) https://BioRender.com/tvyy3wa.

Lasers as desorption ionization sources for mass spectrometry fulfil two functions: (i) ablation of the sample, which in the case of a laser engraver is the primary function, and (ii) ionization. The tested setup, without additional postionization, is expected to follow an REIMS-like mechanism due to the similar instrumental setup used by Schäfer et al., who, however, previously reported that no signal was obtained using REIMS from several different lasers in the visible range.^22^ A laser operating in the blue range at 455 nm was not included in their study; instead, a 532 nm laser was used. As in earlier REIMS studies, the laser was initially directly connected to the MS capillary inlet, using the inherent MS vacuum system to aspirate the generated aerosol.^19,20,22,24−26^ The laser engraver used here originally featured an integrated aerosol removal line to aspirate the produced aerosol and improve the laser functionality and lifetime. We have removed this line as well as the attached fan unit and replaced it with PTFE tubing (1/8 in o.d. 1/16 in i.d.), positioning the opening to approximately 0.5 cm from the sample surface and the laser irradiation point (see Figure 1a). Using this setup, we were able to produce rich, REIMS-like spectral profiles featuring glycerophospholipids, fatty acids, and lyso-glycerophospholipid species in the intermediate mass range (Figures 1b,S1 for a direct comparison of both techniques). Thus, unlike previously concluded by Schäfer et al., a blue range diode laser operating at 455 nm wavelength is capable to be used directly with REIMS to produce molecular ions.

The direct connection to the MS, however, resulted in excessive instrument contamination with pronounced deposits at the atmospheric pressure interface. Thus, in line with more advanced REIMS setups used in intraoperative settings,^20^ we have implemented an aerosol transfer setup featuring a Venturi pump. While the opening of the tubing was still placed in front of the MS capillary inlet, this enabled us to insert a particle collection element directly in front of the MS inlet to capture some of the particulate contamination before it enters the MS. Using this setup, we measured >150 samples per day, before performing preventive cleaning of the API. Further options to improve this setup include positioning the aerosol outlet at a 90° angle to the MS inlet and introduction of isopropanol vapors to avoid contamination buildup.^27^ A further improvement can be achieved through inclusion of a heated collision surface as available in commercial solutions by Waters Corporation (Milford), in the case of skimmer-type APIs.

### Optimization of Laser Parameters

**Figure 2:**
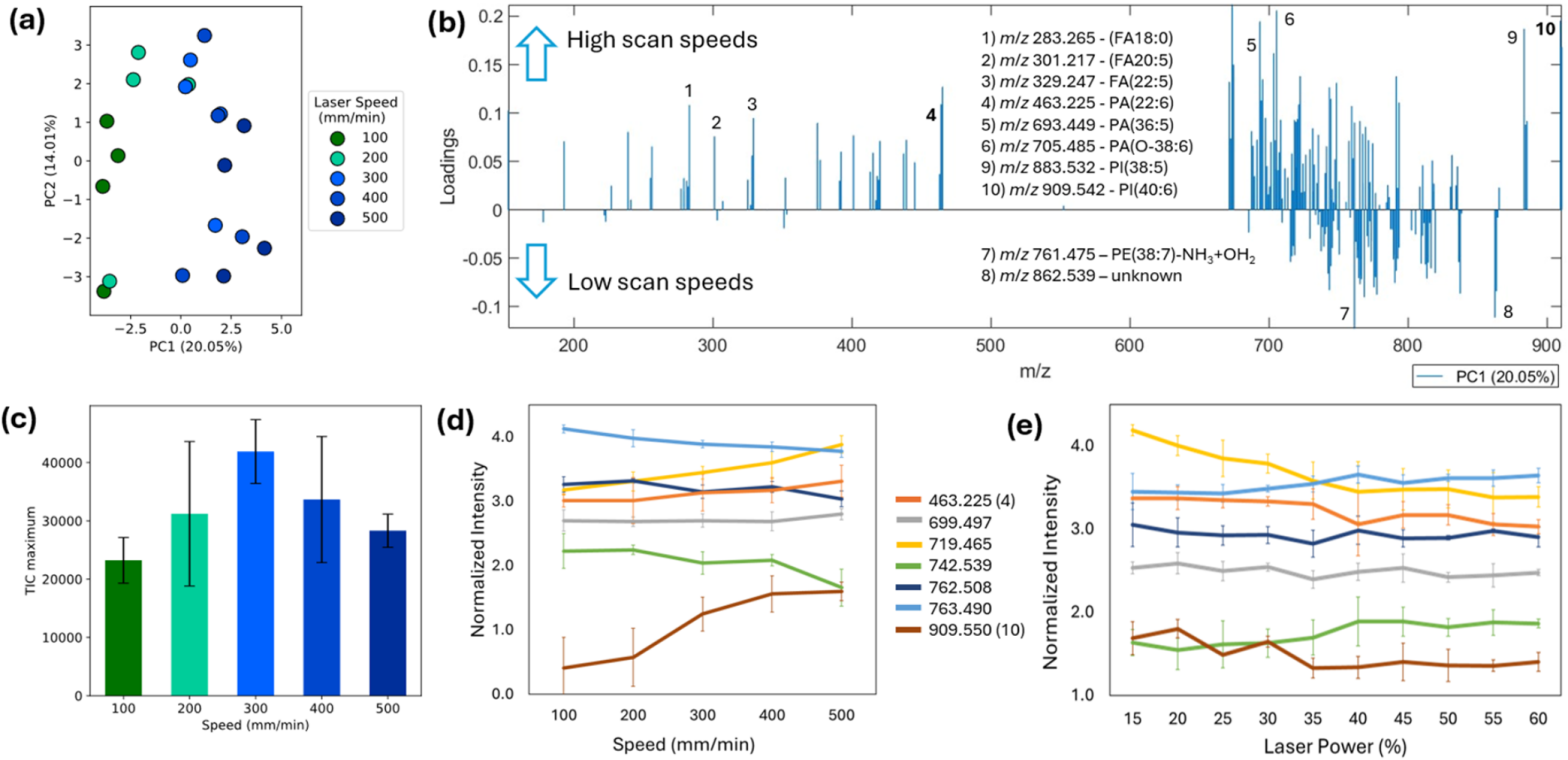
Results of laser optimization studies. (a) PCA of data (*n* = 5 each) recorded at different laser scanning speeds from 100 to 500 mm/min, (b) PC1 loadings from panel (a), (c) TIC plotted as a function of laser scanning speed (*n* = 4 each), and (d, e) intensity trend of selected lipid species as a function of laser speed and laser power for lipid in-source fragment (orange), PE(34:1)–NH_3_ (gray), PA(38:6) (yellow), PC(34:2)–CH_3_ (green), PE(38:6) (dark blue), PE(38:6)–NH_3_+H_2_O (light blue), and PI(40:6) (brown).

For the operation of the laser, the following settings were optimized and tested for their effect on spectral appearance and intensity: (i) Laser scanning speed, (ii) laser power, and (iii) gas pressure at the Venturi pump. The laser focal point was kept constant and controlled according to the manufacturer’s recommendations. Each setting was systematically tested with four replicates on salmon tissue, measured in a randomized order. The laser scanning speed was optimized in the range of 100–500 mm/min. Scanning speed resulted in a systematic change in spectral appearance, as can be seen in the principal component analysis (PCA) scatter plot in Figure 2a, which shows a trend from low to high scanning speeds along the first principal component. As the PC1 loading plot in Figure 2b shows, a general trend toward lower molecular masses can be seen for higher scan speeds. While the majority of lipids increase toward higher scan speeds, individual lipid subsets can be seen decreasing (Figure 2d). This applies to phosphatidylethanolamines (PEs), while phosphatidic acids (PAs) and phosphatidylinositols (PIs) are generally observed to increase. Phosphatidyl glycerols (PGs) and phosphatidylserine (PS) lipids were not among the major detected phospholipid classes. Thus, the optimum laser speed is dependent on lipid class. However, a laser speed of 300 mm/min constitutes a good compromise with the overall highest TIC values (see Figure 2c). A slower laser speed might result in increased energy deposited in each area. This is consistent with several of the increased species under low scan speeds resulting from lipid thermal decomposition reactions, such as methyl group loss of PCs, ammonia loss from PEs, and hydrolysis products of PEs in which the ammonia group is replaced by a hydroxyl group (observable by a shift of +1 Da). These were identified via MS/MS analysis of the fatty acid (FA) composition and the presence of characteristic fragments. These fragments are specifically *m*/*z* 140.996 and *m*/*z* 197.022 for the PE hydrolysis product (PE–NH_3_+OH_2_) and *m*/*z* 168.043 and *m*/*z* 224.069 for the methyl group loss from PC species (PC–CH_3_) (see Figure S2 for suggested structures). A similar trend was observed for the laser power setting and normalized peak intensity ablation (see Figure 2e, performed at 300 mm/min speed setting); however, absolute intensity was generally increasing with increasing laser power (see Figure S3) due to increased material due to the increased tissue ablation. To avoid excessive charring and instrument contamination, we have chosen an intermediate laser power of 25–35%. We have repeated these experiments at 100 mm/min scan speed and found the results to be similar. Venturi gas pressure between the tested input gas pressures of 1–5 bar did not influence the obtained spectra or intensities in a systematic manner (Figure S4).

### Comparison of REIMS vs 455 nm Laser

**Figure 3:**
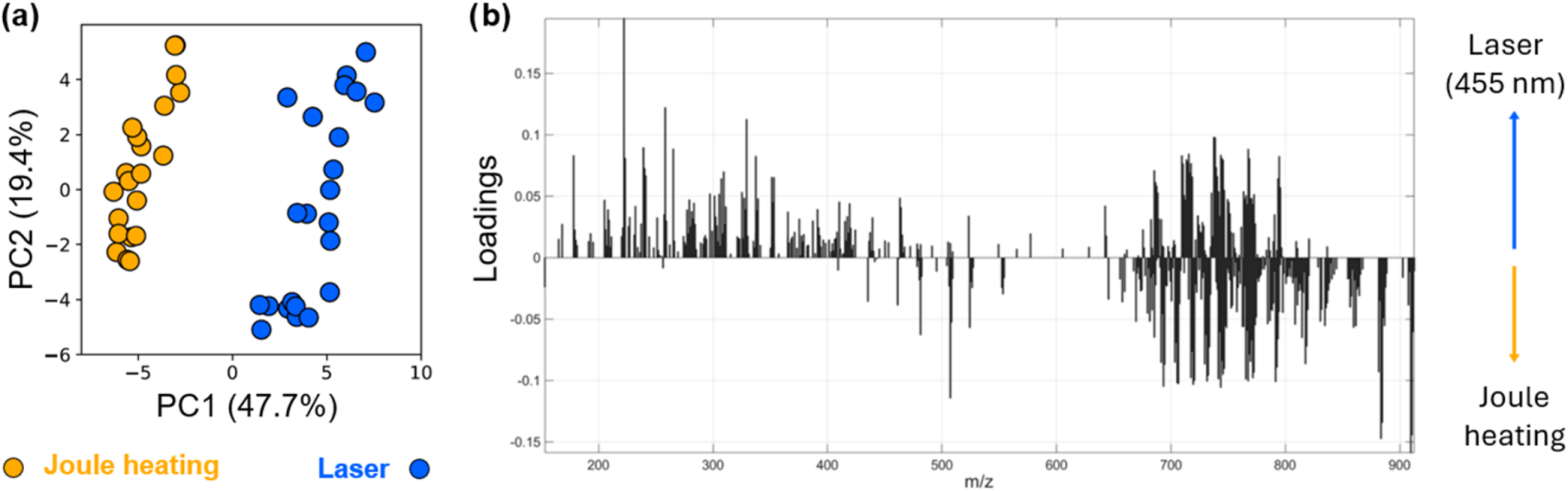
Comparison of data obtained by using Joule heating (conventional REIMS) versus a 455 nm laser engraver. (a) Principal component analysis plot and (b) corresponding loading plots for PC1 of salmon recorded using both setups^26,28^.

Although previously no signal was obtained in LD-REIMS studies using lasers operating in the visible range, we suspect a REIMS-like ionization mechanism for the blue laser setup due to the high similarity in the instrumental setup. Thus, to characterize the spectral overlap between standard REIMS and the laser engraver, we performed REIMS analysis using a monopolar setup with a needle electrode (based on Joule heating) and the blue laser with optimized settings as described above to generate spectral profiles of salmon tissues. REIMS has been previously shown to be a powerful tool to identify fish species, differentiate between their region of origin and even between different modes of fishing (as shown for line-caught vs trawler-caught haddock).^26,28−34^ Both spectra show large similarities (Figure S1) and are dominated by glycerophospholipid (GPL) and FA species (see Figure 1b). The data were subjected to PCA, and a clear separation between Joule heating and laser-derived spectra can be seen along PC1 (Figure 3a). The Joule heating data cloud is observed to be more tightly packed, indicating slightly higher data variation in the spectra acquired using the blue laser. ANOVA was performed to test for systematic, univariate changes in peak abundance between the two methods. Among the most significantly changed peaks between the two modalities, none was completely specific to either Joule heating or the blue laser method, indicating that the same molecular species, including in-source fragments and adducts, are observed in both methods. General awareness is increasing that fragmentation occurs during REIMS analysis, especially for phosphatidylcholines (PCs) and phosphatidylethanolamines (PEs), which are thought to be susceptible to full and partial headgroup loss in REIMS, resulting among others in in-source fragments of the type [PC–NH(CH_3_)_3_]^−^, [PC–CH_3_]^−^, and [PE–NH_3_]^−^.^28^ These might cause interference with other potential lipid species, such as the corresponding PA in the case of full headgroup loss from any other GPL group, as well as PA+C_2_H_2_ in the case of ammonia loss from PEs or partial headgroup loss from PCs (–N(CH_3_)_3_). Thus, in addition to previously published annotations^26,28^ in salmon, we have performed our own fragmentation analysis using MS/MS and used specific headgroup-related fragments (see Figure S2) as well as FA chain composition for GLP annotation.

Among the systematic changes observed between Joule heating and the blue laser, the latter is found to produce higher peaks in the lower molecular mass range (FAs, lyso-lipids/lipid breakdown products), while Joule heating leads to generally higher intensity in the intact GLP range (*m*/*z* 600–900). This is thought to be due to the harsh conditions of the plasma formed at the site of laser irradiation, leading to increased thermal fragments and degradation products. A similar effect was observed by Balog et al. for different generator power settings in Joule heating-based REIMS.^19^ For instance, intact PAs and PEs are generally found higher in conventional REIMS spectra, while several *m*/*z* species, including lipid fragmentation products such as PC–CH_3_ species and PE hydrolysis species (*m*/*z* 715.492, 717.509, 737.476, and 763.490, see Figure S6), are observed at increased abundance using the laser. While these PE hydrolysis species are not unique to the blue laser, they have not been previously reported as annotations in REIMS. PE–NH_3_ (such as *m*/*z* 699.497 and 697.482, PE(34:1)–NH_3_ and PE(34:2)–NH_3_, respectively) species are, however, detected at comparable levels in both laser and Joule heating setups.

Further, mass spectral features at *m*/*z* 391.225, 417.241, 419.257, 437.210, 439.225, and 463.225 are observed with higher abundance using the blue laser engraver (Figure 3b). These are likely derived from GLPs following in-source fragmentation, containing FAs 16:0, 18:1, 18:0, 20:5, 20:4, and 22:6, respectively, as confirmed by the presence of the corresponding fatty acids in the fragmentation spectra (see Figure S5).

Notably, peaks at *m*/*z* 222.088 and 239.114 are both higher in the laser-produced spectra. These are thought to be anserine, an antioxidant that is increased in white muscle of salmonid species,^35^ and an in-source fragment resulting from ammonia loss (mass difference: −17.027 Da), that is also seen in the anserine fragmentation spectra.

### Investigations into REIMS-like Ionization Mechanism

Further common features between standard REIMS using Joule heating and the laser setup are that neither produces any discernible lipid signal from liver and salmon tissue in positive ion mode. Only matrix-assisted REIMS^22^ as well as postionization setups^22,36^ were previously shown to enable analysis in positive ion mode. In REIMS, it is suggested that as a first step thermal desorption occurs and that surface collisions of neutral compound clusters are involved in the ionization process. While this was the rationale for the development of the commercial REIMS interface (available for Waters instruments) that features a heated collision surface following the inlet transfer capillary placed at the opening of the Stepwave ion guide, there is only empirical and nonpublished evidence of this process in skimmer-type atmospheric pressure interfaces (APIs), which are deployed in a range of Thermo Fisher Scientific instruments that were used in many of the early REIMS studies.^19,24^

**Figure 4:**
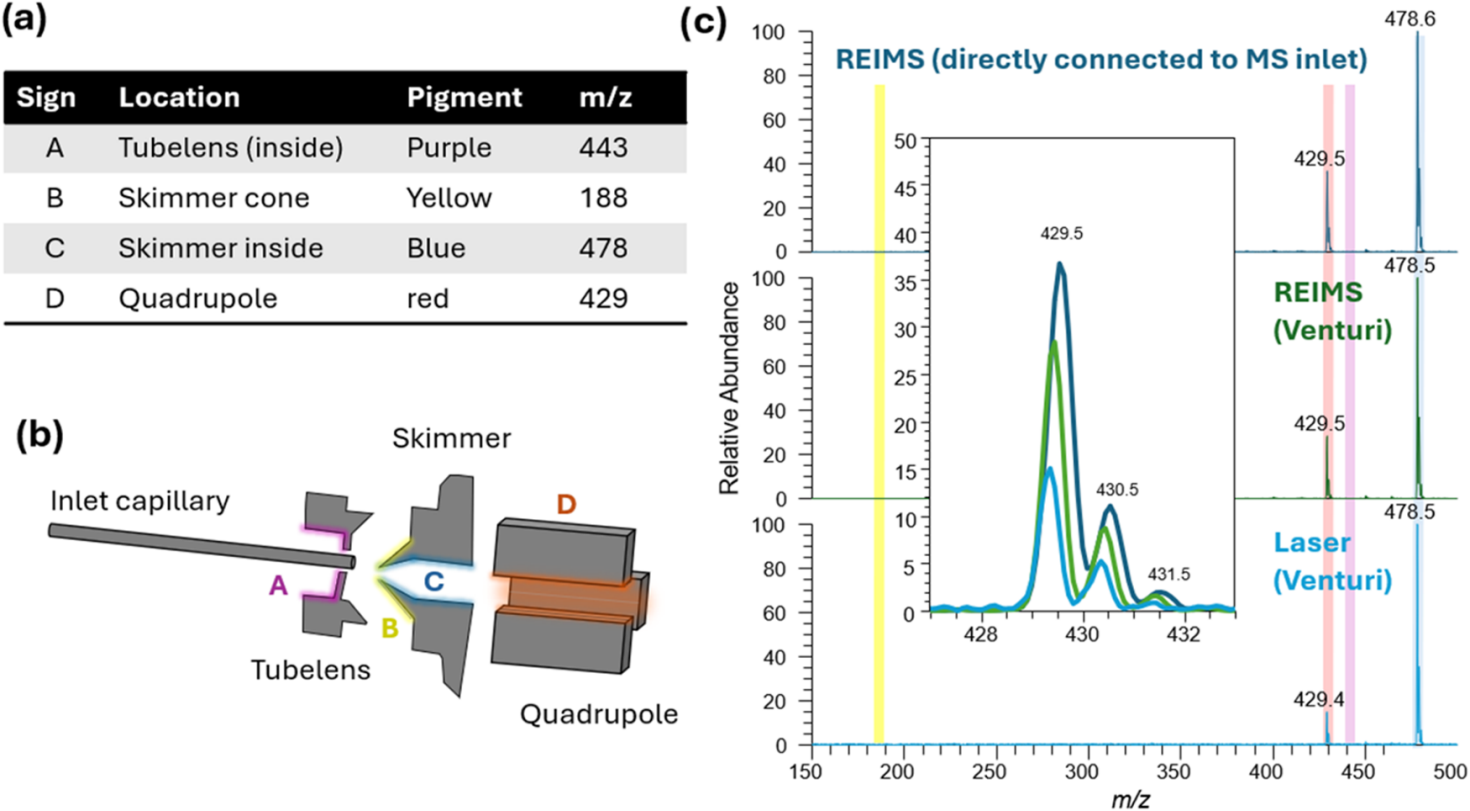
Similar processes occur in the API between REIMS and vis-LD-REIMS. (a) Table showing which pigments were used to color different parts of the API in a Thermo LTQ XL instrument. (b) Scheme API of a Thermo LTQ XL; (c) spectrum obtained in positive ion mode during measurements with REIMS, REIMS with a Venturi setup, and the laser with a Venturi setup.

To further consolidate this hypothesis, unlike in conventional electrospray, no effect of tube lens voltage and capillary voltage setting is observed on the overall spectral intensity in REIMS, suggesting that the formation of individual ions occurs after entering the MS (see Figure S7). This effect was previously reported by Balog^19^ and Günther et al.,^36^ respectively, for Thermo mass spectrometers. To study which surfaces exactly are involved in ion production, we have covered individual surfaces of the API with different easily ionizable analytes, expecting them to desorb and appear in the spectra upon surface collisions. To this end, we have screened several permanent markers for suitability (small overall number of peaks with high intensity) using DESI (see Figure S8) and subsequently deposited these markers onto the different API surfaces of a Thermo LTQ XL: (1) tube Lens, (2) skimmer outer cone, (3) skimmer cone inner surface, and (4) square quadrupole ion guide (see Figure 4a+b). The instrument was then re-evacuated, and measurements were performed using standard monopolar REIMS and the blue laser engraver setup. In all cases, only pigments that originated from the inside of the skimmer and the square quadrupole ion guide were observed, while no signal originated from the tube lens and the outer skimmer cone. While the outer skimmer cone is the main site of instrument contamination (showing macroscopic buildup of deposits), any ions resulting from these collisions on the outer skimmer surface do not enter the skimmer and subsequent ion optics. We hypothesize that the contamination originating from the outer skimmer cone results from large aerosol droplets with a relatively low mobility.

Smaller aerosol droplets, however, can likely follow the gas flow and enter the opening in the skimmer cone, where subsequently collisions occur on the inside of the skimmer as well as on the surfaces of the square quadrupole rods. This can be seen for all setups tested. While the absolute intensities of the different dyes cannot be compared directly, the ratio between the dyes on both surfaces is a more robust indicator of changes in the contribution of different ion optical parts to these surface collisions. A decrease in the ratio of quadrupole to skimmer collisions from 0.4 in standard REIMS to 0.3 in the REIMS Venturi setup was observed (see Figure 4c), the latter being associated with the smaller droplet size due to the additional pneumatic assistance. This ratio ultimately decreased to 0.15 in the laser setup. This suggests an increase in the contribution of skimmer collisions over quadrupole collisions as the aerosol droplet size decreases, likely caused by the decreased travel distance of smaller droplets in lower pressure environments. These data suggest a similar ion formation process in conventional REIMS and the laser setup, positioning this blue laser engraver as the first reported visible laser light variant of LD-REIMS.

### Capability to Differentiate Organic from Conventional Farmed Salmon

**Figure 5:**
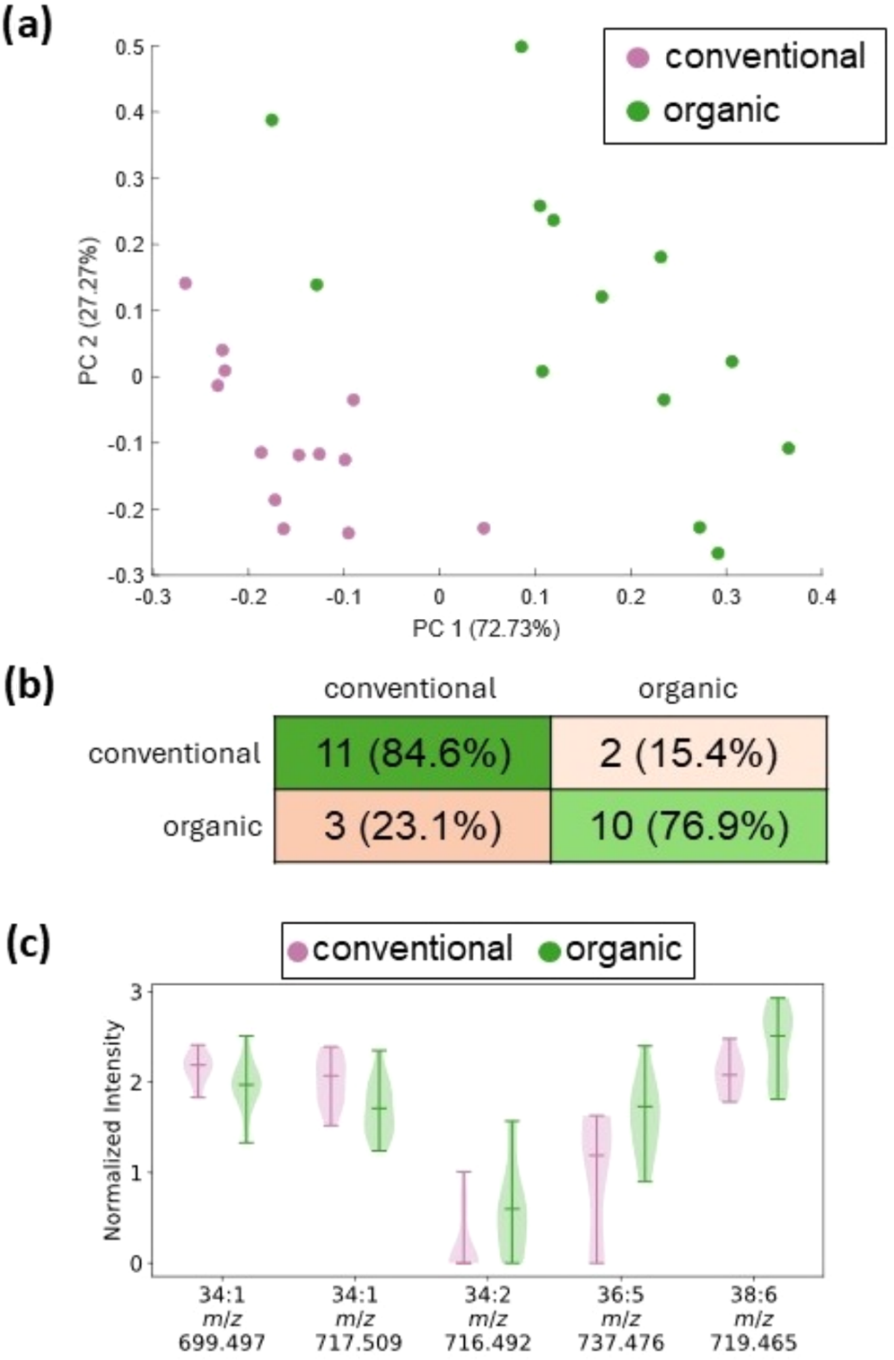
Partial least-squares discriminant analysis (PLS-DA) results of spectra from the blue light laser. (a) Scores plot, (b) confusion matrix with two components after leave-sample-out cross-validation, and (c) ratio of intensity of lipids between salmon tissue from organic or conventional farming as a function of lipid chain length and unsaturation for the lipid classes PAs and PEs.

Salmon is one of the most consumed fish in Europe, as either wild salmon or farmed salmon. For the latter, consumers can choose between wild salmon or conventional and organic aquaculture. As organic aquaculture is associated with higher costs than conventional farming methods, food fraud by mislabeling is a common issue. To test the ability of the blue laser setup to be used for this type of food fraud application, we applied it to the analysis of salmon from Norwegian aquaculture from both conventional and organic farming methods (*n* = 13 each). The data were then subjected to a supervised analysis using partial least-squares discriminant analysis (PLS-DA). The scores plot (Figure 5a) shows that a separation into two distinct groups is visible. The classification model was evaluated using leave-sample-out cross-validation. The performance of the model can be assessed with a confusion matrix (Figure 5b), giving a precision of 0.786 for conventional farming (recall = 0.846) and 0.833 for organic farming (recall = 0.769), which demonstrates a promising overall performance, which might further improve with an increased size of the sample set as the capacity of multivariate models generally improves with the size of the training data.

In addition, Lasso regression^37^ was performed to test for specific markers for either conventionally or organically farmed salmon. In total, nine peaks were found to be specific for organically farmed salmon, with seven of them being increased in this class. The seven markers that were found to be specific for the conventional salmon overlapped with the results for organic salmon but with the opposite sign of the coefficient (Table S4). Except for the marker at *m*/*z* 777.565 being tentatively identified by mass as PG(36:0), none of the markers could be annotated. However, excellent performance of the model was observed to classify organic vs conventional farming methods with ROC values of 0.9941 and 1.0 for conventional and organic groups, respectively (Figure S9).

Interestingly, the recorded data show that a trend toward relatively higher abundance of lipids with a higher degree of unsaturation is visible for both the PA and the PE lipid classes, as identified by calculating the intensity ratio from the mean intensities in conventional and organic salmon for selected, annotated lipid species. Mass spectral features at *m*/*z* 716.492, 719.465, and 737.476 are observed with higher abundance in the fish from organic farming, while lipids with single desaturation appear higher in conventional farmed salmon (Figure 5c). The observation of different levels of certain lipids in conventionally and organically farmed salmon is consistent with studies from Trocino et al. in sea bass, who showed that levels of certain polyunsaturated FA differ between fish from conventional and organic rearing, especially the n-6 and n-3 PUFAs. The difference was mainly attributed to the ratio of vegetable oil and fish oil in the diet of the fish.^38,39^

## Conclusions

We have shown that a commercially available laser engraver can be used as a cheap, automated ambient ionization source that leads to direct ion formation without additional postionization steps, relying instead on an REIMS-like ion formation process. This proves that, unlike previously thought, visible light lasers are suitable energy devices for REIMS analysis The higher degree of in-source fragmentation observed with the blue laser complicates the sharing of spectral databases between the different energy devices that can be used for REIMS analysis. Thus, the acquisition of spectral databases is recommended with the respective energy device used. While this makes GLP identification more difficult, highlighting the definitive need for MS/MS analysis to identify the lipids present, it does not necessarily interfere with the qualitative profiling analysis of tissues. Specific headgroup fragments were identified and proven to be useful in identifying different lipid fragmentation products. We have shown that this setup is a promising platform for assessing food fraud, such as the differentiation of organic farming from conventional farming. Unique advantages of this setup are the ready-to-use nature and intuitive operation through apps, as well as the large range of motion up to 40 × 40 cm^2^. We plan to develop this setup further to improve aerosol transfer and decrease instrument contamination through a combination with a dedicated REIMS API as well as optimize the setup for imaging applications of samples that are too large for standard MSI setups.

## Supporting Information Available

Tables detailing sample sets used for Laser vs REIMS and organic vs conventional comparisons; comparison of spectra REIMS vs Laser; table of tentative lipid annotations; characteristic fragments for lipid in-source fragmentation products; effect of laser power setting and Venturi gas pressure on spectra; suggested in source fragmentation mechanism; representative fragmentation spectra of PE hydrolysis products; effect of ion optical elements on TIC; DESI-MS spectra of permanent markers; results associated with LASSO regression (PDF)
