# Supplementary material for: Commercially Available Blue Diode Laser Engraver Operating at 455 nm as an Affordable LD-REIMS Ionization Source
Source: Anal Chem. 2025 Apr 30;97(18):9961–9. doi: 10.1021/acs.analchem.5c00724 (PMC12079636; doi:10.1021/acs.analchem.5c00724)
Supplement: Supplementary file 1 — ac5c00724_si_001.pdf [file ac5c00724_si_001.pdf]

Supplementary Material

A commercially available blue diode laser engraver operating at 455 nm as affordable LD-REIMS ionization source

Prisca Weider‡, Daniel Heffernan‡, Min Qiu, Marco Klein, Carl Witthöft, Wei Chen, Nicole Strittmatter*

Affiliations:

[1] Technical University of Munich, Department of Biosciences, School of Natural Sciences, 85748 Garching b. München, Germany

*corresponding author: Nicole Strittmatter, Nicole.strittmatter@tum.de, T: +49 89 28913321

‡authors contributed equally.

Contents: Tables detailing sample sets used for Laser vs REIMS and organic vs conventional comparisons, comparison of spectra REIMS vs Laser, table of tentative lipid annotations, characteristic fragments for lipid in source fragmentation products, effect of laser power setting and Venturi gas pressure on spectra, suggested in source fragmentation mechanism, representative fragmentation spectra of PE hydrolysis products, effect of ion optical elements on TIC, DESI-MS spectra of permanent markers, results associated with LASSO regression.

Table S1. Salmon samples used for the comparison of Joule heating vs 455 nm laser.

Producer	Country of Origin	Farming	Condition at Purchase	Date of Catch
Berida	Norway	Conventional	Frozen	07.02.2023
Costa	Norway	Conventional	Frozen	18.09.2023
Costa	Faroe	Conventional	Frozen	03.02.2023
deutsche see	Norway	Conventional	Frozen	20.09.2023
Edeka Bio	Norway	Organic	Frozen	24.01.2024
Edeka Freshfood Counter	Norway	Conventional	Fresh	n/a
Fischerstolz	Norway	Conventional	Fresh	18.03.2024
Fischerstolz Bio Organic	Norway	Organic	Fresh	18.03.2024
followfood	Norway	Organic	Frozen	11.07.2023
Golden Seafood	Norway	Conventional	Frozen	21.12.2023
Gourmet Finest Cuisine	Norway	Conventional	Fresh	n/a
Gut und Günstig	Norway	Conventional	Frozen	02.01.2024
Gutbio	Norway	Organic	Frozen	30.11.2023
Icewind	Norway	Conventional	Frozen	15.11.2022
K classic	Norway	Conventional	Frozen	13.07.2023
Kaufland Bio	Norway	Organic	Frozen	30.05.2023
Kaufland Freshfood Counter	Norway	Conventional	Fresh	n/a
Lachshaus Riga	Ireland	Organic	Fresh	20.03.2024
Naturgut	Norway	Organic	Frozen	17.11.2023
Oceansea	Norway	Conventional	Frozen	12.05.2023
Oceansea	Faroe	Conventional	Frozen	15.02.2024
REWE Bio	Norway	Organic	Frozen	19.02.2024
REWE Bio	Norway	Organic	Frozen	20.02.2024

Table S2. Salmon samples used for the comparison of farming conditions (organic vs conventionally farmed).

Producer	Country of Origin	Farming	Condition at Purchase	Date of Catch
Berida	Norway	Conventional	frozen	07.02.2023
Berida	Norway	Conventional	frozen	05.04.2024
Costa	Norway	Conventional	frozen	18.09.2023
deutsche see	Norway	Conventional	frozen	20.09.2023
deutsche see	Norway	Organic	frozen	21.05.2024
deutsche see	Norway	Conventional	frozen	26.10.2023
Edeka Bio	Norway	Organic	frozen	24.01.2024
Edeka Bio	Norway	Organic	frozen	16.03.2024
followfish	Norway	Organic	frozen	11.07.2023
followfood	Norway	Organic	frozen	11.07.2023
Golden Seafood	Norway	Conventional	frozen	21.12.2023
Golden Seafood	Norway	Conventional	frozen	24.01.2024
Gut und Günstig	Norway	Conventional	frozen	02.01.2024
Gut und Günstig	Norway	Conventional	frozen	19.01.2024
Gutbio	Norway	Organic	frozen	30.11.2023
gutbio	Norway	Organic	frozen	09.04.2024
Icewind	Norway	Conventional	frozen	15.11.2022
Ja!	Norway	Conventional	frozen	25.05.2024
K classic	Norway	Conventional	frozen	13.07.2023
Kaufland Bio	Norway	Organic	frozen	30.05.2023
Naturgut	Norway	Organic	frozen	17.11.2023
Naturgut	Norway	Organic	frozen	01.02.2024
Oceansea	Norway	Conventional	frozen	12.05.2023
Rewe Bio	Norway	Organic	frozen	19.02.2024
Rewe Bio	Norway	Organic	frozen	20.02.2024
Rewe Bio	Norway	Organic	frozen	10.05.2024

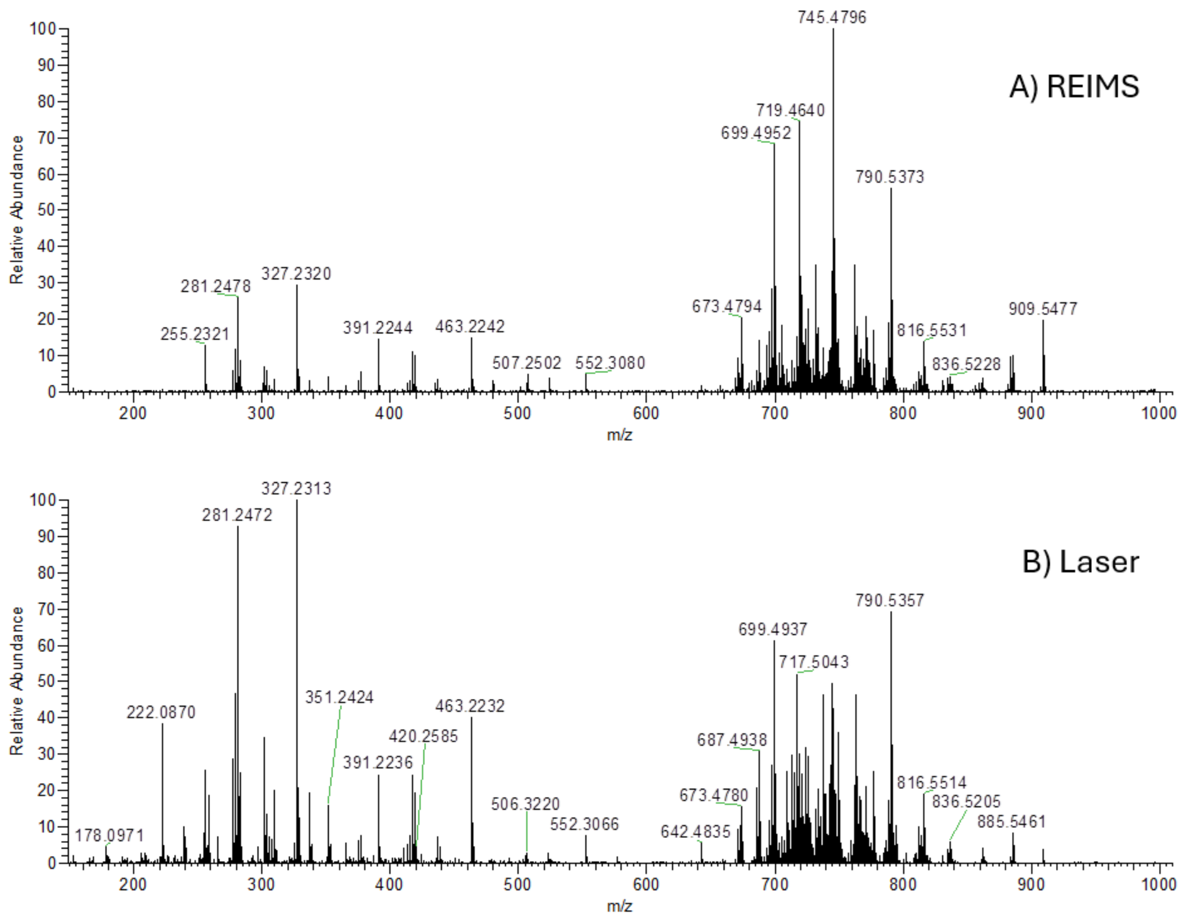

Figure S1. Direct comparison of spectra for the same salmon specimen (Edeka Bio) for A) REIMS using conventional Joule heating and B) Laser operating at 455 nm, respectively.

Table S3. Lipid IDs as identified from our own MS/MS measurements. Further annotations can be found in references *Song et al.*[1] and *Yin et al.*[2]

**m/z measured**	**Species**	**Chain composition**
673.480	[PA-H]$^-$	34:1
697.482	[PE-NH$_3$]$^-$/[PC-N(CH$_3$)$_3$-H]$^-$	34:2
699.497	[PE-NH$_3$]$^-$/[PC-N(CH$_3$)$_3$-H]$^-$	34:1
715.492	[PE-NH$_3$+OH$_2$-H]$^-$	34:2
717.509	[PE-NH$_3$+OH$_2$-H]$^-$	34:1
719.465	[PA-H]$^-$	38:6
731.502	[PA-H]$^-$	O-40:7
737.476	[PE-NH$_3$+OH$_2$-H]$^-$	36:5
742.539	[PC-CH$_3$]$^-$	34:2
744.555	[PC-CH$_3$]$^-$	36:1/34:1
745.481	[PA-H]$^-$/[PE-NH$_3$]$^-$/[PC-N(CH$_3$)$_3$-H]$^-$	40:7/38:6
747.518	[PG-H]$^-$	34:1
749.512	[PA-H]$^-$	40:5
762.508	[PE-H]-	38:8
763.490	[PE-NH$_3$+OH$_2$-H]$^-$	38:6
764.524	[PE-H]-/[PC-CH$_3$]$^-$	38:5/36:5
766.538	[PE-H]-/[PC-CH$_3$]$^-$	38:4/36:4
770.570	[PC-CH$_3$]$^-$	36:2 (18:1/18:1)
771.495	[PE-NH$_3$]$^-$/[PC-N(CH$_3$)$_3$-H]$^-$	40:7
772.529	[PE-H]-/[PC-CH$_3$]$^-$	O-42:8/O-40:8
774.542	[PE-H]-/[PC-CH$_3$]$^-$	O-42:7/O-40:7
776.560	[PE-H]-	O-40:6
786.508	[PC-CH$_3$]$^-$	38:8
788.523	[PC-CH$_3$]$^-$	38:7
789.506	[PE-NH$_3$+OH$_2$-H]$^-$	40:7
790.540	[PC-CH$_3$]$^-$	40:6/38:6
792.552	[PE-H]-/[PC-CH$_3$]$^-$	40:5/38:5
805.482	[PI-H]$^-$	32:2
816.555	[PE-H]-/[PC-CH$_3$]$^-$	42:7/40:7
833.518	[PI-H]$^-$	34:2
835.526	[PI-H]$^-$	34:1
857.518	[PI-H]$^-$	36:4
859.531	[PI-H]$^-$	36:3
861.550	[PI-H]$^-$	36:2
883.532	[PI-H]$^-$	38:5
885.547	[PI-H]$^-$	38:4
909.550	[PI-H]$^-$	40:6

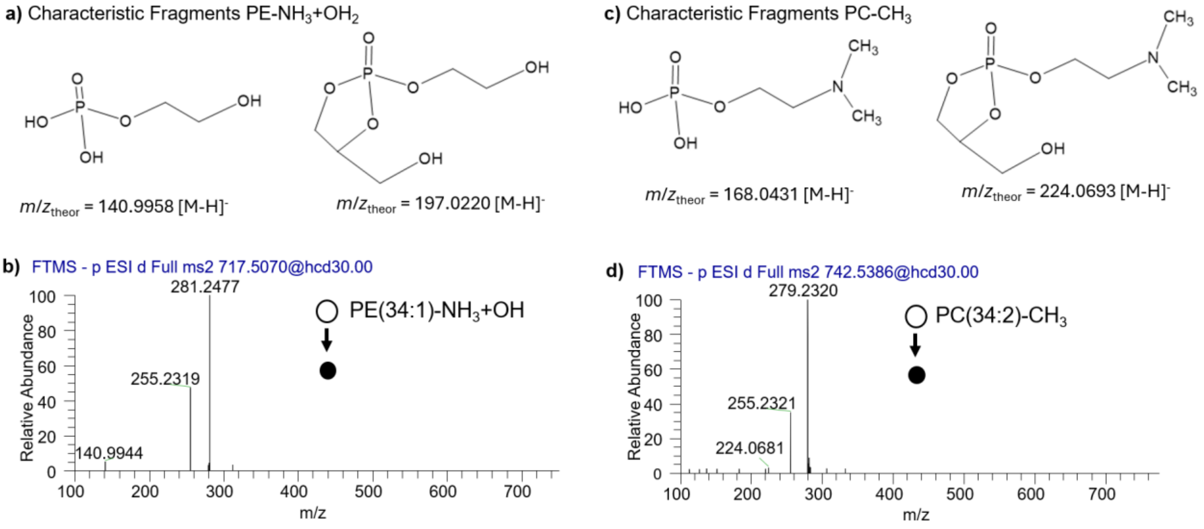

Figure S2. a+c) Characteristic fragments identified for lipid degradation products of the type $PE\text{-}NH_3\text{+}OH_2$ and $PC\text{-}CH_3$ with example fragmentation spectra displaying these fragments (b+d). The equivalent intact PE fragments (no hydrolysis) can be found at *m/z* 140.0118 and 196.0380, respectively.

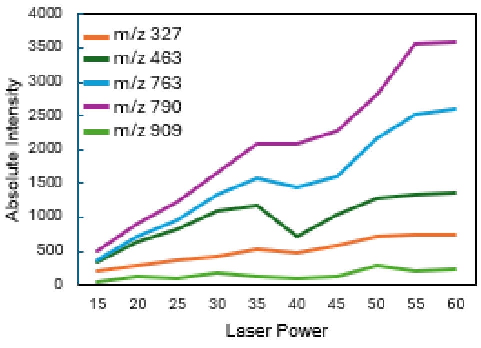

Figure S3. Absolute peak intensity as a function of laser power setting.

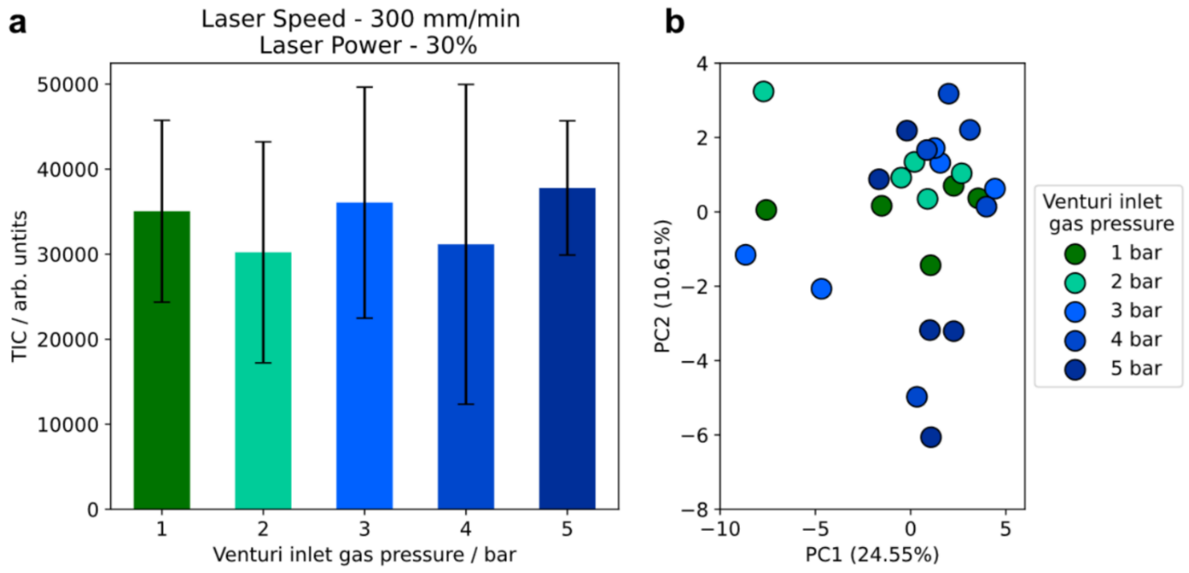

Figure S4. Effect of Venturi gas pressure on a) TIC and b) spectral appearance as seen in a PCA.

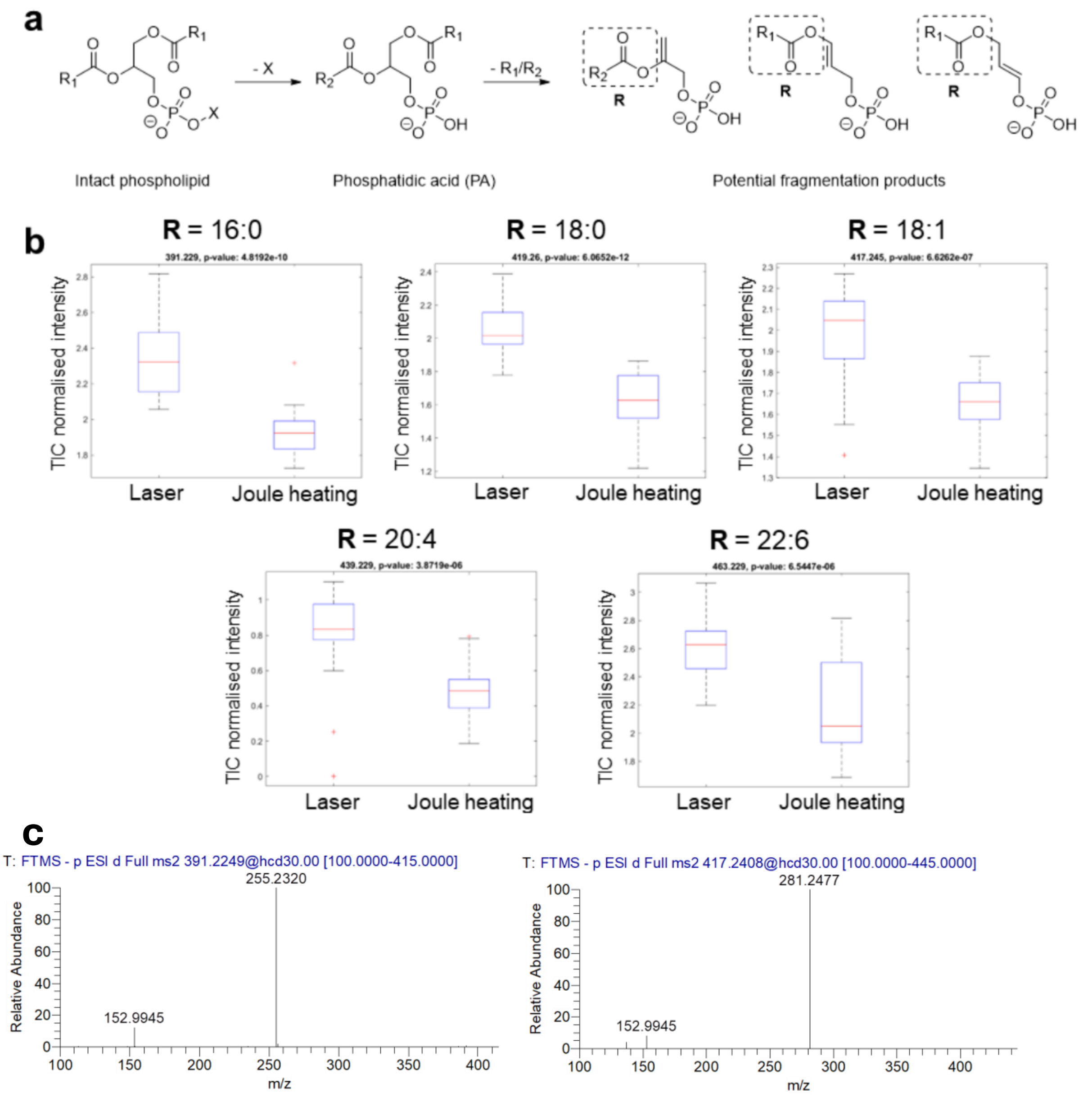

Figure S5. Suggested in-source fragmentation mechanism resulting in fragment ions in the lyso-lipid mass range (*m/z* 400-500). c) Fragmentation spectra of exemplary species *m/z* 391 and *m/z* 417 resulting in C16:0 and C18:1 fatty acids and cyclic glycerophosphatidyl fragment, respectively.

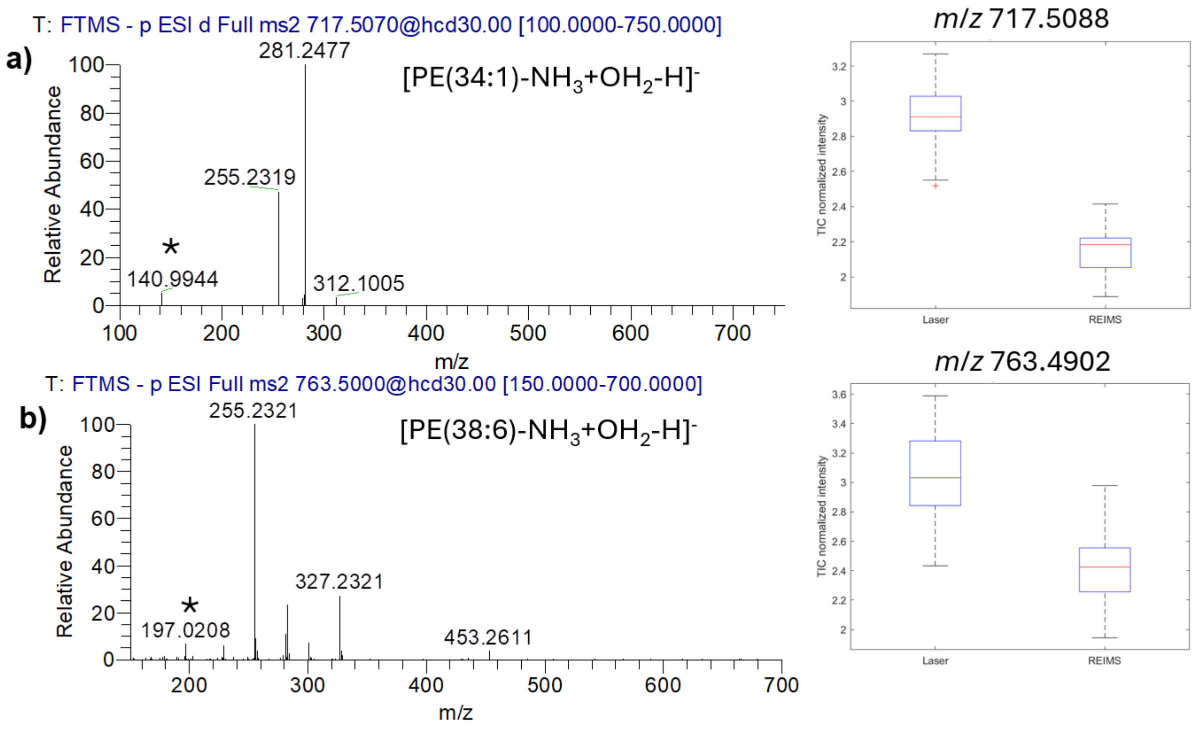

Figure S6. Representative fragmentation spectra and boxplots in laser vs REIMS analysis for $[PE-NH_3+OH_2-H]^-$ species for a) $[PE(34:1)-NH_3+OH_2-H]^-$ and b) $[PE(38:6)-NH_3+OH_2-H]^-$. Asterix marks characteristic fragments from Figure S2.

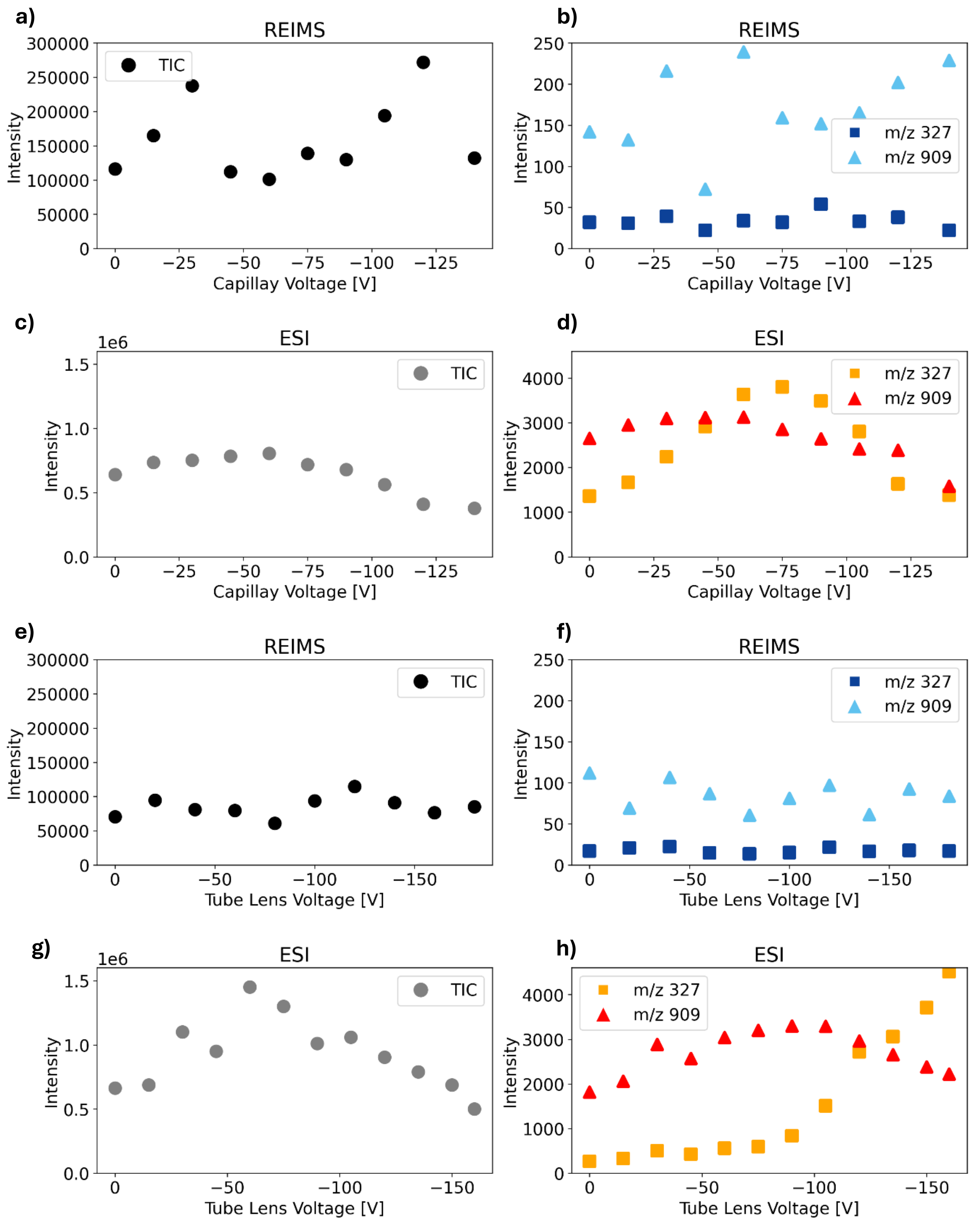

Figure S7. TIC (left panel) and selected ion traces (right panel) showing the influence of capillary voltage (a-d) and tube lens voltage (e-h) on spectra acquired using monopolar Joule heating REIMS and Electrospray on a LTQ XL (Thermo Scientific) instrument. Selected ions are *m/z* 327.233 (identified as FA22:6) and *m/z* 909.550 (identified as PI(40:6) to exemplify the effect on high and low mass range analytes, respectively.

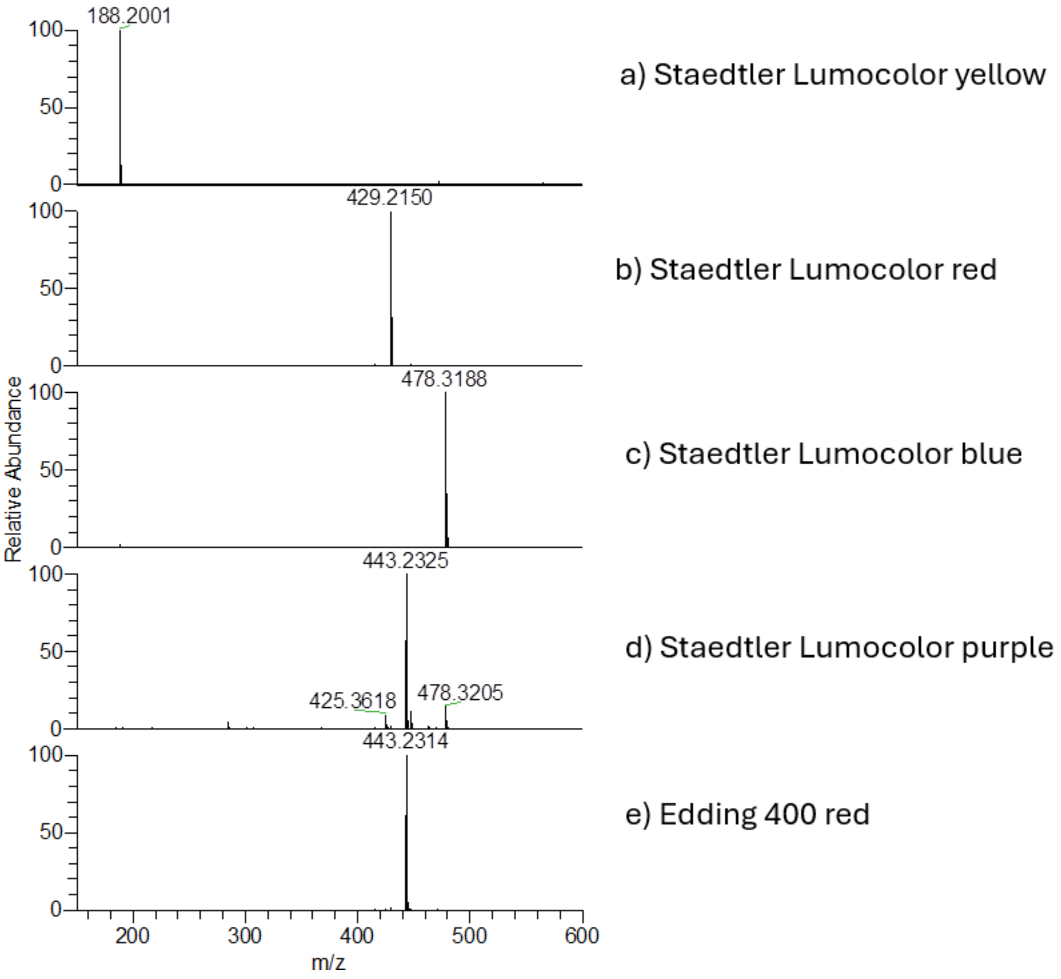

Figure S8. DESI-MS data of different permanent markers on glass slide. a-d) Staedtler Lumocolor and e) Edding 400. d and e) are deployed instead of each other. *m/z* 478 (Nile Blue 7) in blue can receive interference of d), however, interference can be excluded if *m/z* 443 (Rhodamine isomer) is not detected.

Table S4: Peaks identified as specific in lasso regression within the groups of conventional and organic salmon. Plots of coefficient path, cross-validated fits and the ROC curves for each group can be found in **Figure S8**.

	Lasso Coefficient			
m/z	Conventional	Organic	p-Value	Attribution
232.113		-	0.292	
303.248	-	+	0.259	
308.165	-	+	0.175	
313.168	-	+	0.025	
341.210		+	0.135	
353.263	-	+	0.676	
392.232	-	+	0.152	
764.497	-	+	0.280	
777.565	+	-	0.409	PG36:0

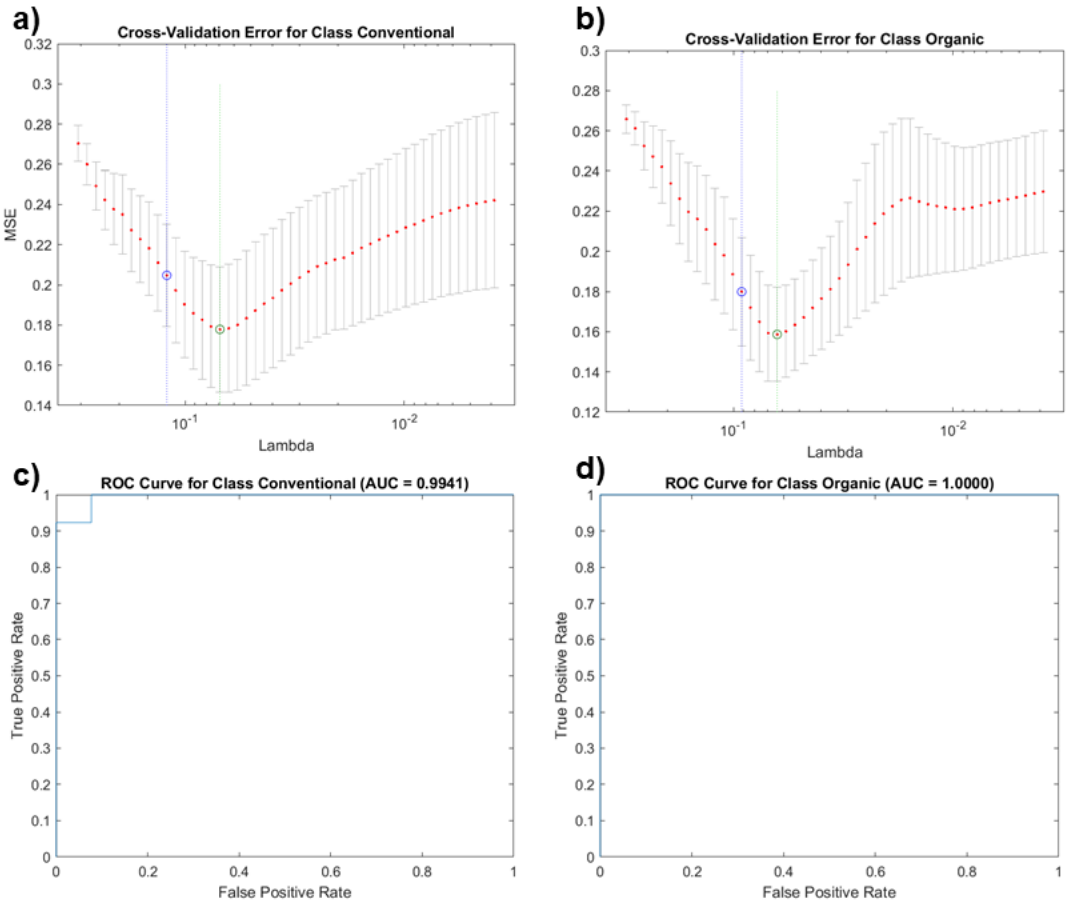

Figure S9. Cross-validated fits and ROC curves for the group of a)+c) conventionally farmed and b)+d) organically farmed salmon from the lasso regression.

References

(1) Song, G.; Zhang, M.; Zhang, Y.; Wang, H.; Li, S.; Dai, Z.; Shen, Q. In Situ Method for Real-Time Discriminating Salmon and Rainbow Trout without Sample Preparation Using iKnife and Rapid Evaporative Ionization Mass Spectrometry-Based Lipidomics. *J. Agric. Food Chem.* **2019**, *67* (16), 4679–4688. https://doi.org/10.1021/acs.jafc.9b00751.

(2) Yin, X.; Wang, H.; Lu, W.; Ge, L.; Cui, Y.; Zhao, Q.; Liang, J.; Shen, Q.; Liu, A.; Xue, J. Evaluation of Lipid Oxidation Characteristics in Salmon after Simulation of Cold Chain Interruption Using Rapid Evaporation Ionization Mass Spectrometry. *J. Agric. Food Chem.* **2024**, *72* (2), 1391–1404. https://doi.org/10.1021/acs.jafc.3c07423.
